# Modulation of Elementary Calcium Release Mediates a Transition from Puffs to Waves in an IP_3_R Cluster Model

**DOI:** 10.1371/journal.pcbi.1003965

**Published:** 2015-01-08

**Authors:** Martin Rückl, Ian Parker, Jonathan S. Marchant, Chamakuri Nagaiah, Friedrich W. Johenning, Sten Rüdiger

**Affiliations:** 1 Institut für Physik, Humboldt-Universität zu Berlin, Berlin, Germany; 2 Departments of Neurobiology and Behavior, Physiology and Biophysics, University of California, Irvine, Irvine, California, United States of America; 3 Department of Pharmacology, University of Minnesota, Minneapolis, Minnesota, United States of America; 4 Johann Radon Institute for Computational and Applied Mathematics, Austrian Academy of Sciences, Linz, Austria; 5 Neuroscience Research Center, Charité-Universitätsmedizin Berlin, Berlin, Germany; Johns Hopkins University, United States of America

## Abstract

The oscillating concentration of intracellular calcium is one of the most important examples for collective dynamics in cell biology. Localized releases of calcium through clusters of inositol 1,4,5-trisphosphate receptor channels constitute elementary signals called calcium puffs. Coupling by diffusing calcium leads to global releases and waves, but the exact mechanism of inter-cluster coupling and triggering of waves is unknown. To elucidate the relation of puffs and waves, we here model a cluster of IP_3_R channels using a gating scheme with variable non-equilibrium IP_3_ binding. Hybrid stochastic and deterministic simulations show that puffs are not stereotyped events of constant duration but are sensitive to stimulation strength and residual calcium. For increasing IP_3_ concentration, the release events become modulated at a timescale of minutes, with repetitive wave-like releases interspersed with several puffs. This modulation is consistent with experimental observations we present, including refractoriness and increase of puff frequency during the inter-wave interval. Our results suggest that waves are established by a random but time-modulated appearance of sustained release events, which have a high potential to trigger and synchronize activity throughout the cell.

## Author Summary

Intracellular calcium oscillations and waves are paramount cellular signals. The frequency of global release events regulates, for example, expression of genes. Knowledge about the mechanism of oscillations and the factors that determine their frequency is crucial when aiming at the control of downstream processes. Many experimental and modeling studies have demonstrated that a calcium cycle consists of both deterministic and stochastic components, but the respective mechanisms are under debate. Here we aim to clarify both components by analyzing calcium release in *Xenopus* oocytes and a computational model for a cluster of IP_3_ receptor channels. Just as in calcium fluorescence traces, in the computed sequences some of the events are prolonged releases lasting for several seconds. We find that synchronized unbinding and rebinding of IP_3_ cause this modulation in time. Our experimental and computational data show agreement in many properties including wave period, extended refractoriness, and release amplitude. Our analysis suggests that global calcium concentrations are stochastically oscillating because of a modulated but random appearance of high-release events. Thus our approach integrates both deterministic properties and stochasticity of waves, and reveals key control parameters of calcium oscillations.

## Introduction

Transient and repetitive increases in the concentration of cytosolic Ca^2+^ are ubiquitous chemical cues in a cell. They are crucial for neuronal adaptation, cell growth and myocyte function, to name a few examples. The formation of complex intracellular release patterns plays an important role in cell communication, since Ca^2+^ achieves its functional specificity by differential signaling in space, time, and amplitude [1]. It is therefore a purpose of numerous research studies to understand the systemic generation of cytosolic Ca^2+^ signals [2].

In many non-excitable cells, increasing the concentration of inositol 1,4,5-trisphosphate (IP_3_) triggers Ca^2+^ release from the endoplasmic reticulum (ER) by activating intracellular IP_3_ receptor (IP_3_R) channels in the ER membrane. Opening of IP_3_R channels is induced by the binding of IP_3_ and Ca^2+^ [3] and therefore Ca^2+^ released from open channels and diffusing through the cell can recruit further IP_3_R channels to open. If the IP_3_ stimulation is modest, Ca^2+^ is released from spatially confined clusters of intracellular Ca^2+^ channels [4]. Molecular interactions within a cluster lead to coherent opening of its channels and result in local elementary events called puffs [5]–[7]. The small number of IP_3_R channels involved in a puff (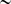
10) and the random appearance of puffs suggest their spontaneous generation by microscopic fluctuations, which has been related to classical excitability in activator-inhibitor systems [8], [9]. For larger stimulation, however, Ca^2+^ forms more regular spatio-temporal waves or whole-cell oscillations involving release by multiple clusters and many channels [10]–[12].

The transition from small localized release to Ca^2+^ waves across cells has been addressed by a large number of experimental and theoretical studies, see e.g. [8], [13]–[20]. The interest particularly concerns the mechanism for the generation of waves or global oscillations, because it controls the oscillation frequency, which is known to regulate important cellular functions [21]. Ca^2+^ diffusion coupling is responsible for communication between clusters [11], [22], [23], thus mediating the synchronization of clusters into oscillations and the propagation of waves, but it is less clear what are the respective roles of increased IP_3_ stimulation and Ca^2+^ diffusion in the puff-to-wave transition. Enhanced synchronization with increasing [IP_3_] can in principle be mediated by two IP_3_ dependent scenarios. In a first scenario (i) the excitability of clusters grows with [IP_3_] so that a given amount of Ca^2+^, e.g. diffusing from an active cluster, triggers puffs more frequently. Cluster excitability is expected to increase with rising IP_3_ concentration because the number of channels with bound IP_3_ increases. In a second scenario (ii) the amount of Ca^2+^ diffusing from an active cluster to clusters in its proximity increases with [IP_3_] for instance because of larger release current or longer release. In both scenarios the likelihood of propagation of activity from cluster to cluster increases with [IP_3_].

Recent modeling has often assumed that puffs are stereotyped events with a relatively constant amplitude and life time. Therefore, several studies focused on scenario (i), which implicates that for larger stimulation the frequency of puffs increases. Furthermore, it has been suggested that the occurrence of a supercritical number of puffs during a short time interval leads to nucleation of a wave from a cellular subdomain [24]–[26]. Taken together, it follows that wave nucleations occur more often when the puff frequency increases, i.e., for larger stimulation and excitability. Several aspects of intracellular Ca^2+^ waves are in very good accord with this explanation of the puff-to-wave transition, particularly the large variability in the inter wave interval (IWI) [26]. However, other important features of global releases, including their long lifetime and the extended refractoriness, are difficult to accommodate with present models and a realistic biomathematical model showing wave nucleation and the mentioned features remains to be devised. Here we pursue a new model of intracellular Ca^2+^ release that exhibits the puff-to-wave transition and the randomness of global releases and, in addition, displays the previously unexplained facets of Ca^2+^ waves.

Our model is based on excitable dynamics of a single cluster that goes beyond the activator-inhibitor schemes. To achieve its complexity, the model incorporates the possibility of slowly decaying release from a cluster [27]. This slow release phase involves a residual domain, i.e., Ca^2+^ which is transiently present in the cluster domain after a channel has closed. Then, the residual domain provokes perpetual reopening of channels in the cluster. This particularly happens when the timescale of recovery from inhibition, 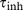, is shorter than the typical decay time of the residual domain, 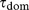, so that channels lose inhibiting Ca^2+^ before [Ca^2+^] is below activating levels. It was found in [27], that in this case termination of release necessitates a further negative regulation, different from inhibition, and it was shown that this “inactivation” can be provided by transient unbinding of IP_3_ from the receptor. This previously overlooked effect originates from allosteric coupling between inhibiting Ca^2+^ binding and IP_3_ binding, which is a hallmark of IP_3_R gating [28].

To address the puff-to-wave transition in the context of complex cluster dynamics, we here analyze in detail the time courses of release in their dependence on IP_3_ concentration. We are using the most complete computer simulation of intracluster [Ca^2+^] that is presently available. It allows us to incorporate the effects of residual Ca^2+^ transients on channel opening. We find that with increased IP_3_ concentration the number of IP_3_ molecules bound to receptors (excitability, scenario i) and the amplitude of release from a cluster only slightly increase, while the lifetime of the signal increases dramatically (scenario ii). This growing event lifetime can be related to the former finding of perpetual re-opening due to residual Ca^2+^ in the domain. Crucially, the mean lifetime increases because of the appearance of a subset of sustained release events at increased [IP_3_], which thus constitutes a stimulation-dependent transition in the release pattern. We further find that this effect pertains to the increased variability in the number of channels that have IP_3_ bound. It is then likely, that, for a subset of events, the lifetime of residual domains increases because of larger total flux from the cluster, while 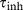 typically remains constant [29]. As a result, for sufficiently increased stimulation 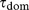 is larger than 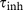 and release becomes extended for several seconds until termination by IP_3_ loss.

The transition to a qualitatively altered regime in our cluster model generates surprisingly many dynamical and statistical features that are in excellent agreement with the experimental descriptions of global releases. The modification in release shape and duration can be related to a study of temporal profiles of Ca^2+^ liberation in *Xenopus* oocytes in response to [IP_3_] step increases, which reported two distinct phases [30]. At moderate [IP_3_], fluorescence recordings exhibit a fast but short release flux. For larger [IP_3_], however, Ca^2+^ efflux involves first a time course similar to that observed for small [IP_3_] followed by a second phase of small amplitude release lasting several seconds. The fast and slow processes correspond well to the dynamics in the excitable system described by our model.

The prolongation of release in the simulations and the comparison to experiments therefore hint at scenario (ii), i.e., it is the magnitude of Ca^2+^ release that predominantly grows with stimulation. This leads to a new explanation for the puff-to-wave transition, in which generation of waves emerges from the complex and stochastic excitable dynamics of a single cluster. The fact that sustained events in our computations resemble waves in their fluorescence traces suggests to identify these events with global release. In our simulations we find accumulating amounts of Ca^2+^ in the local domain during sustained events, which endows them with higher potential to establish synchronized release of multiple clusters. Thus, although a Ca^2+^ wave is a spatially organized event involving many clusters, the capacity of triggering or participating in a wave is a distinctive feature of long-lasting events in our single cluster model, which, in this sense, qualifies them as global events or waves. Propagation of waves, from our point of view, is probabilistic and depends on the positions and distances of adjacent channel clusters, but it is fundamentally the change in release time course that primes the channels for global release and that enables the synchronous opening of clusters.

As a distinctive feature of large- [IP_3_] simulations we find that the IP_3_ unbindings and rebindings become very frequent for long-lasting, wave-like events. During release many channels may lose IP_3_ synchronously, while subsequently to the termination of release they rebind IP_3_. These dynamics of IP_3_ binding/unbinding, synchronized to the appearance of wave-like release, causes a modulation of the number of activatable channels over timescales of minutes. The frequency of puffs occurring between waves is then strongly modulated and exhibits the refractoriness and increase of puff frequency known from experiments [11], [31]. To the best of our knowledge, this crucial feature of Ca^2+^ waves has not been addressed in modeling studies so far.

It is important to note that puffs in our simulations cannot be compared directly to those of experiments with EGTA-loaded cells [32], [33]. Residual Ca^2+^ domains are suppressed by EGTA buffer, so that sustained release may not be observed in this setup [27], [34]. Appearance of long-lasting events in the present simulations without exogenous buffer is therefore not in contradiction to the short puffs occurring in experiments for large [IP_3_] and with EGTA loading. EGTA thus prevents residual Ca^2+^ domains, appearance of prolonged release and, consequently, unbinding of IP_3_. Nevertheless, occasional stochastic IP_3_ binding/unbinding may occur also in this situation and lead to puff variability. This finding may also explain part of the fluctuating puff amplitudes found in recent experiments with EGTA loaded SH-SY5Y cells [33] but will not be discussed in the present study.

The article is organized as follows: We first describe the basic components of our model, consisting of Markovian gating of the IP_3_R channels and a reaction-diffusion system for the evolution of cytosolic concentrations of Ca^2+^ and Ca^2+^ binding proteins. We also summarize our numerical method. In contrast to our earlier publication [27], we here employ the finite element method (FEM) for three-dimensional concentration fields and a hybrid scheme to couple local Ca^2+^ concentrations and channel gating states [35]. We then discuss our main findings based on simulation traces that typically cover several thousands of seconds simulation time. Here, we describe the distributions of event lifetimes and their dependence on IP_3_ concentration. We then analyze the role of IP_3_ unbinding for the lifetimes and discuss the stochastic variability. Finally, we suggest that the large number of long-lasting events for large IP_3_ concentration can be related to the frequency of global events. We here define a global event by an individual cluster's capacity to trigger adjacent clusters. In the final part of the paper, we draw a relation of such events to wave generation and dynamics in the inter-wave period and compare our results to experimental recordings.

## Materials and Methods

### Ethics statement

Experiments were performed on immature oocytes obtained from *Xenopus* laevis as described previously [4], [13]. Frogs were anaesthetized by immersion in a 0.15% aqueous solution of MS-222 (3-aminobenzoic acid ethyl ester) for 15 min, and small pieces of ovary removed by surgery following procedures approved under UCI IACUC (University of California, Irvine, Institutional Animal Care and Use Committee) protocol 1998-1337.

### Modeling the intracellular Ca^2+^ dynamics

In this section we will describe the components of our mathematical model for channel gating, ion and buffer diffusion, and chemical reactions. The model consists of a Markov chain for IP_3_R channel states [36] and partial differential equations for spatial concentration fields (Ca^2+^ and buffers). For numerical implementation, the two stochastic and deterministic paradigms are coupled by the hybrid method introduced in [35].

#### Stochastic model of IP_3_R channel gating

**Figure 1:**
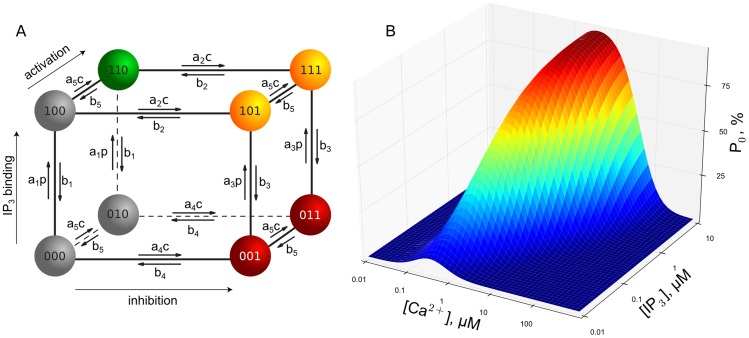
**(A) Gating scheme of the modified DYK model used to model the states of a single subunit.** The transition rates are determined by [Ca^2+^] and [IP_3_] here denoted by 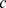 and 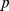, respectively, and the 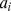 and 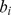 as given in Table 1. **(B) Steady state open probability of the modified DYK model for a single channel.** For increasing [IP_3_], the open probability increases and the maximum of the open probability shifts to higher [Ca^2+^].

The open and close dynamics of IP_3_R channels are incorporated via a modified DeYoung-Keizer (DYK) model [38], [39]. Here, an IP_3_R channel consists of four identical subunits, where each subunit possesses three different binding sites: An activating site for Ca^2+^, an inhibiting Ca^2+^ site, and an IP_3_ binding site. Hence, a subunit can undergo transitions between eight different states X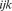 (see Fig. 1 A). The index 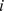 indicates the state of the IP_3_ site, 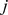 the one of the activating Ca^2+^ site and 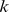 the state of the inhibiting Ca^2+^ site. An index is 1 if Ca^2+^ or IP_3_ are bound and 0 if not. A channel is defined to be open if at least three of the four subunits are in the state X_110_ [15]. The parameters defining the transition rates of the model were fitted to patch-clamp data for type 1 IP_3_R channels in [28], [40], [41] and are given in Table 1. These rates have been chosen at values similar to our earlier publication [27] except for 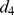 (and consequently 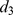), which has been adjusted by a factor of about 3 to reflect a large shift in open probability in dependence on [IP_3_]. In order to obey detailed balance, these parameters have to satisfy the following condition:
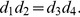
(1)This relation is crucial for the unbinding dynamics of IP_3_ that we will report on below [27].

**Table 1:** Channel gating parameters 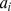, 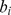 and the dissociation coefficient 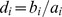 of the modified DYK model.

	*i*	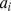 in 1/(µMs)	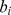 in 1/s	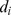 in µM
IP_3_ while not inhibited	1	0.20	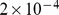	0.001
Inhibition with IP_3_	2	0.02	1.56	78
IP_3_ while inhibited	3	0.40	0.80	2
Inhibition without IP_3_	4	0.10	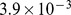	0.039
Activation	5	100	25	0.25

It is important to note, that the binding of IP_3_ depends on whether a subunit is inhibited or not: on the right hand side of the DYK cube (Fig. 1 A), the equilibrium [IP_3_] (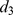) is higher than on the left hand side (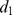, see Table 1). Hence, it is more likely that a subunit will unbind IP_3_ if it is inhibited for sufficiently long times. This behavior is related to the shift of the right branch in the open probability graph with increasing [IP_3_] (i.e., 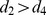, see Fig. 1 B) and the detailed balance condition (1). If two or more subunits of a channel lose IP_3_, the channel is rendered *unactivatable*, because the remaining subunits cannot lead to a channel opening according to the criterion given above. *Vice versa*, we term a channel as *activatable*, if at least three of its subunits have bound IP_3_. While the loss of IP_3_ is promoted by inhibition, it still has to be distinguished from normal inhibition due to its slower time scale (see below). Furthermore, note that in our definition, an inhibited channel might still be activatable (e.g., two subunits in X_110_ and two subunits in X_111_).

To combine the stochastic channel events with deterministic reaction-diffusion processes of Ca^2+^ and buffers, we employ the hybrid algorithm described in [35]. To calculate the binding rates of activating Ca^2+^, the [Ca^2+^] is evaluated at the center of the channel pore.

#### Reaction-diffusion model

The interaction of Ca^2+^ with buffers and the diffusion of Ca^2+^ and buffers are treated as a system of coupled reaction-diffusion equations. In the following, 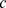 and 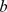 will denote the free cytosolic [Ca^2+^] and bound buffer concentration, respectively. The indices 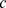 and 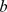 will also be used to distinguish between different other species-specific parameters. Assuming simple reaction kinetics this leads to the following system of PDEs:
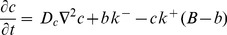
(2)




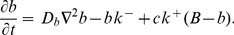
(3)

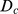
, 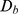 and 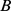 denote the diffusion coefficients of free Ca^2+^, bound buffer and the total buffer concentration, respectively.

Reaction and diffusion take place in a cuboidal domain 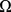 with boundary 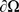. The lower plane of the cuboid 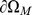 represents the ER membrane, on which a single cluster consisting of 16 IP_3_R -channels is located. The channels are arranged in a 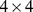 regular grid. The spatial extent of the entire cuboid is 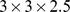 µm^3^. The boundary conditions for 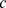 and 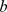 on the ER membrane are:
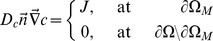
(4)




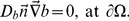
(5)Here, 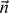 denotes the outer normal vector of the boundary of the domain 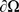. 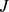 describes the flux through the membrane and comprises three contributions:
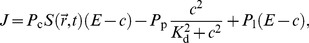
(6)where 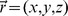 denotes the spatial position on the membrane and 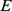 is the [Ca^2+^] in the ER lumen. In the following we assume 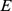 to be constant.

The first term in Eq. (6) models flux through the IP_3_R -channels from the ER to the cytosol. This term is controlled by the channel state through the factor 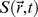, which is defined by:
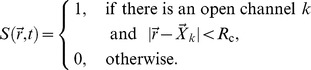
(7)Here, 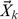 and 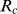 denote the location of channel 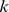 (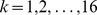) and the channel pore radius, respectively. The total current 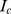 through the pore is determined by 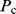 and the finite extent of the pore by:
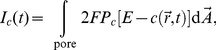
(8)where 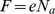 is the Faraday constant. To calculate the flux coefficient 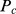 from the channel current 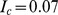 pA [42]–[45] we approximate the integral in Eq. (8) by 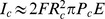. For the chosen parameters (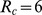 nm), the [Ca^2+^] at an open channel pore reaches peak values up to 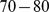 µM. Hence, with 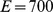 µM the above approximation is roughly satisfied. Since smaller pore radii (and hence larger 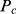) achieve larger peak pore concentrations, a dependency of the dynamics of the DYK model on the channel pore radius arises. To avoid this problem, we do not drive the DYK model with the concentration measured at the channel pore, but universally set it to 150 µM, for open channels. Further, this method allows us to significantly reduce computation time since one can choose larger pore radii and hence a lower grid resolution for the FEM.

The second term in Eq. (6) models SERCA pumps. Standard models as the one in Eq. (6) are of Hill equation type with Hill coefficient 2 [46]. 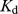 is the dissociation constant of the pumps. The maximal pump current, 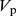, was estimated to 10–16 µM s^−1^ [47]. Note that this number is based on a volume source and needs to be cast into a flux through a boundary by multiplying the volume current by the domain extension 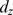. This results in units of moles per surface area and time, as is required for the boundary flux.

The last term in Eq. (6) models a small leakage of Ca^2+^ from the luminal to cytosolic domain. Besides its possible physiological relevance, it here also serves to balance the system at rest state, i.e., it compensates the SERCA pumps when there are no open channels. Hence, to achieve a resting [Ca^2+^] of a few tens of nM (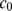) in the cytosolic domain and few hundreds of µM in the ER (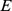), equating the two last terms of Eq. (6) provides a dependence of 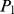 on 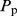:
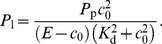
(9)


The PDEs (2, 3) were solved on a conforming, locally refined grid using the finite element library DUNE [48], [49]. Parallelization for 4–16 CPUs was achieved by domain decomposition [37]. Time integration of the resulting system of coupled ODEs was performed with a linear implicit three stage Runge-Kutta algorithm using parameters known as ROWDA3 [50].

**Figure 2:**
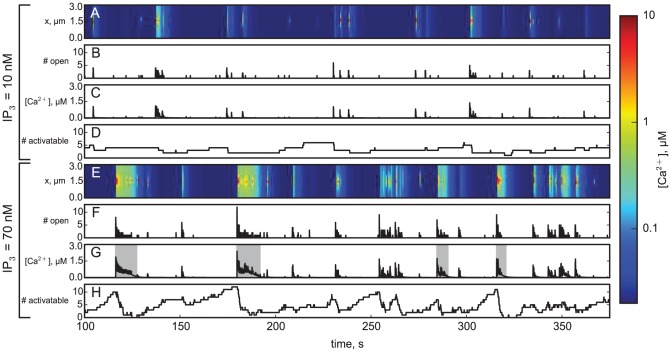
Exemplary simulations for [IP_3_] = 10 nM (A-D) and [IP_3_] = 70 nM (E-H). In A and E the local [Ca^2+^] ‘line scans’ along a line on the ER membrane running through the cluster's center is shown. Warmer colors correspond to larger Ca^2+^ concentration and indicate the opening of channels. Because the line does not directly intersect with a channel pore, the nanodomain structure around a channel is not fully visible. The corresponding number of open channels is shown in B and F. C and G display the average concentration in the cluster vicinity (500 nm box), D and H show the number of activatable channels, i.e., the number of channels that have bound IP_3_ to at least three of their subunits.

To check whether the domain size is sufficiently large, i.e. whether the noflux boundary conditions would significantly affect intra-cluster [Ca^2+^] evolution, we compared simulations of one event (first event shown in Fig. 2 G) with simulations of the same openings and closings in a much larger domain (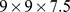 µm^3^). In these simulations (data not shown), we did not observe a significant effect of domain size on intra-cluster [Ca^2+^] and hence conclude that the noflux boundary conditions are a reasonable choice.

### Experimental Ca^2+^ imaging in oocytes

Experimental results on Ca^2+^ imaging of puffs and waves in *Xenopus* oocytes are previously unpublished data acquired during experiments described in [11]. Full experimental methods are given in that paper.

## Results

The system of Eqs. (2, 3) was solved for various values of IP_3_ concentration. The typical simulation interval was set to 2500–3000 s. Because larger [IP_3_] leads to higher channel activity, generally richer statistics are produced with increasing [IP_3_]. Values of parameters introduced in the preceding section are given in Table 2.

**Table 2:** Model parameters of the reaction-diffusion equations.

Parameter	Symbol	Value	Unit
Diffusion coefficient	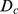	223 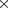 10^6^	nm^2^/s
Cytosolic resting Ca^2+^	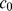	0.02	µM
ER resting Ca^2+^	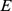	700	µM
Diffusion coefficient	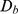	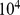	nm^2^/s
Total concentration	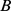	200	µM
On-rate	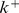	150	1/(µMs)
Off-rate	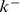	300	1/s
IP_3_R pore radius	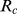	6	nm
Channel flux coefficient	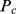	4.58 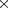 10^6^	nm/s
Channel current	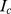	0.07	pA
SERCA pump coefficient	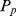	20,000	nm/s
SERCA pump dissociation	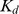	0.05	µM
Leak flux coefficient	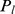	903	nm/s
Domain size	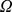	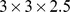	µm ^3^
# of IP_3_R channels	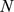	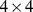	1
inter channel distance	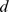	120	nm

Fig. 2 shows representative traces for two simulations, one for low (10 nM, A-D) and one for high (70 nM, E-H) [IP_3_]. Fig. 2 A and E display evolutions of ‘line scans’ through the midpoint of the cluster directly at the membrane, while B and F present the evolution of the number of open channels in the cluster. C and G show the spatial average of the Ca^2+^ concentration in a box containing the cluster vicinity defined by dimensions 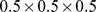 µm^3^ and centered around the cluster. Finally D and H present the evolution of the number of activatable channels in the cluster. Initial conditions of channels of each run were chosen randomly from a steady state regime with low excitability (i.e. high [Ca^2+^]). Transient intervals of 100 s were excluded from all statistics below.

### Event lifetime

To investigate the collective behavior of the IP_3_R channels, we group synchronous channel openings in *collective events*. We define a collective event based on intervals, during which the spatially averaged [Ca^2+^] in the cluster vicinity (500 nm box) exceeds a threshold value of 0.1 µM. All events containing only a single channel opening were filtered out. Using the spatial average of [Ca^2+^] to define a collective event serves to facilitate comparison to experiments on Ca^2+^ puffs and waves in cells with dye buffer, where the number of open channels underlying a release event is not directly known.

Following our event definition, we determine its lifetime or duration from the interval in which the Ca^2+^ concentration exceeds 0.1 µM. Simulations as those in Fig. 2 show that for low [IP_3_] we find short events lasting up to one or two seconds at most. The average duration of release events for [IP_3_] = 10 nM is about 0.5 s, roughly equal to the duration of puffs in experiments on *Xenopus* oocytes [10]. However, for high [IP_3_] we can observe both the short fast-decaying events as well as events characterized by sustained release lasting up to 10 s, akin to release waves observed in the same cell type.

**Figure 3:**
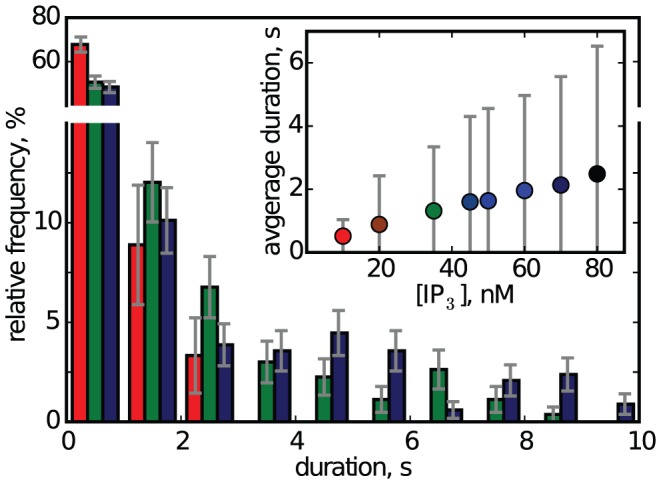
Distribution of event durations for [IP_3_] of 10 nM (red bars), 35 nM (green bars) and 70 nM (blue bars). For [IP_3_] = 10 nM only 3 of 90 events lasted longer than 2 s (3.3%). For [IP_3_] = 20 nM only 10 of 166 events lasted longer than 3 s (6.0%). For [IP_3_] = 80 nM, 72 of 309 events lasted longer than 3 s (23%). Error bars denote the sampling error of the histogram. The inset shows the average event duration and its standard deviation.

Fig. 3 shows the distribution of event durations for different [IP_3_], as well as the average event duration depending on [IP_3_]. For all IP_3_ concentrations, the majority of events (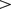 60%) was shorter than one second. However, while for low [IP_3_] all events are shorter than 3 s, for increasing [IP_3_] the distribution develops a wide shoulder. This results in a qualitative change in the distribution accompanied by increased average and increased variance of event durations (Fig. 3 inset). Interestingly, models of release termination by inhibiting Ca^2+^ binding would suggest that larger release amplitudes could only accelerate termination [42]. However, in Fig. 3 we observe both, larger amplitudes and lifetimes, at higher [IP_3_]. To understand this behavior we will now study the IP_3_ binding to the receptors.

### IP_3_ binding variability and impact on release time course

A notable feature of the simulations in Fig. 2 is that the number of activatable channels (i.e. channels that have bound a sufficient amount of IP_3_ to be able to open) is fluctuating both for low (D) and for high (H) IP_3_ concentration. Unbinding of IP_3_ during release was first observed in a model of sustained Ca^2+^ release from IP_3_R channel clusters [27]. Note that the IP_3_ concentrations used in the prior and in the present work exceed the value of the dissociation constant 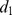 by far (see Table 1) and hence saturated IP_3_ binding sites, i.e., 16 activatable channels, could be expected.

**Figure 4:**
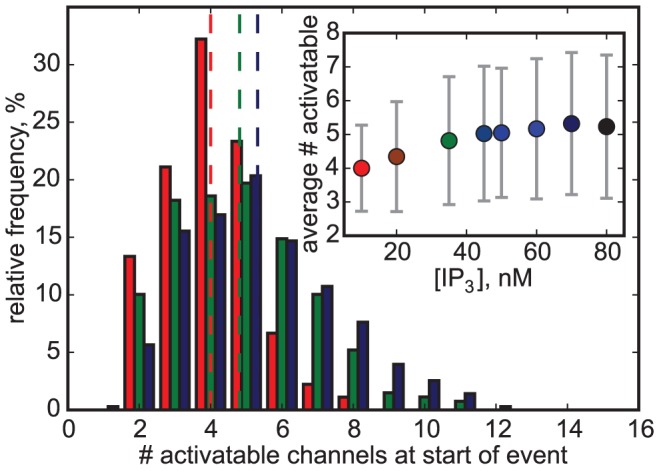
Distribution of the number of activatable channels at the beginning of the events for [IP_3_] = 10 nM (red bars), 35 nM (green bars) and 75 nM (blue bars). Dashed lines mark the mean value. Inset: The average number of activatable channels at the beginning of an event depends on [IP_3_]. Vertical bars are standard deviations.

We will now discuss how the impact of varying IP_3_ concentration on event lifetime is mediated by the dynamics of IP_3_ binding and unbinding, i.e. the dynamics of the number of activatable channels. Events with a higher number of participating channels will generally last longer (see below). A simple approach to elucidate this relation is to count the number of activatable channels *at the beginning* of each event. The distribution of this number of activatable channels is shown for three different IP_3_ concentrations in Fig. 4. Here we can find similar features as before: for increasing [IP_3_] the histogram gets skewed to the right leading to a much increased shoulder. Both the average and the standard deviation of the distribution increase with [IP_3_] (inset). However, the increase of the average number of activatable channels is relatively small, rising from four channels at [IP_3_] = 10 nM to about five channels at 80 nM. On the other hand, the variability doubles for the same range of [IP_3_]. Thus, it is plausible, that the increased variability determines the appearance of extended events.

What causes the increase of variance in the number of activatable channels with increasing [IP_3_]? This effect can be understood with the help of Fig. 2, D and H. First, the number of activatable channels generally decreases during release because of dissociation of IP_3_, and it does so with much larger magnitude for long events found at large [IP_3_] (see Fig. 2 H). Then, after event termination, the cluster is slowly reactivated by IP_3_ rebinding. The resulting rise in the number of activatable channels can be understood as steadily increasing cluster excitability. Hence, at some point, random fluctuations will trigger a new event, resetting some of the channels back to an unactivatable state and closing the circle. While for low [IP_3_], the speed of reactivation is too slow to accumulate a high number of activatable channels, the higher reactivation speed for large [IP_3_] sometimes allows to reach a nearly fully activatable cluster and thus causes the large variability.

**Figure 5:**
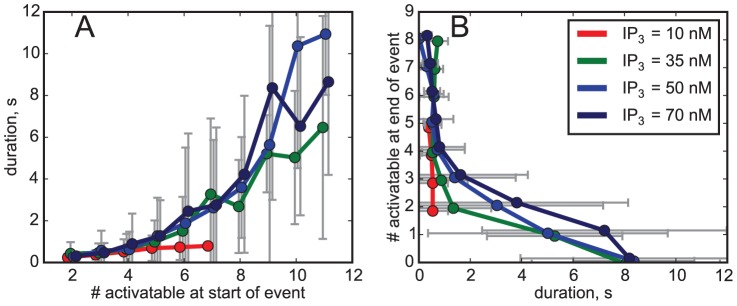
**(A) Event duration depends on the number of activatable channels *at the beginning* of an event.** While low numbers of activatable channels very likely will lead to short events, large numbers do not necessarily lead to long events. **(B) The number of activatable channels at the end of an event as a function of the duration.** Bars denote standard deviations. Absent bars indicate single observations.

Fig. 5 A shows that there is indeed a strong correlation between the number of activatable channels and the event duration. Events starting with only a low number (below 4 or 5) of activatable channels reliably terminate fast. For an intermediate number of activatable channels (6 to 9), there still is a strong stochastic variability of event durations. Finally, for a sufficiently high number almost all events last longer than a few seconds. In other words: a high number of activatable channels is necessary to produce a long event. In the intermediate regime (6 to 9 activatable channels), the time course of the events furthermore depends on the details of the channels' states, e.g. whether activatable channels have 3 or 4 subunits with bound IP_3_ (data not shown).

Taken together, these findings suggest that the increase of release lifetime seen in Fig. 3 is primarily mediated by the increase of variability for larger [IP_3_], and not by the increase of mean number of activatable channels (Fig. 4). The nonlinear increase of duration with the number of activatable channels leads to a substantial increase of lifetime for higher [IP_3_].

We may also ask for the reason of the substantial loss of IP_3_ for some release events. If the number of channels opening synchronously is large, termination by inhibitory Ca^2+^ binding (i.e. subunit transitions from X_110_ to X_111_) is often incomplete, mostly because the residual Ca^2+^ remaining locally after each channel closing is large enough to reopen the channel [27]. The timescale for recovering from inhibition, 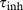, is given by the inverse of the rate 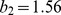 s^−1^: 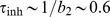 s. Hence, during a single long-lasting event (i.e. longer than 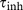), a channel can easily switch back and forth between inhibited, open and resting states. Because inhibition does not guarantee termination for those events, inactivation of channels by IP_3_ dissociation is needed. This can clearly be seen from Fig. 5 B. The long-lasting events will result in a cluster configuration where only a few or even no channel at all remain activatable at the end of release events.

For small [IP_3_], however, the short event durations leave less probability for IP_3_ unbinding. Thus, those short events are a consequence of sufficient inhibition and little reopening probability, because with a smaller number of open channels less Ca^2+^ is extruded and residual Ca^2+^ domains are smaller [34].

### Classification of events into puffs and waves

The experimental observations of puffs and waves, as well as the diversity of the simulated release events for high [IP_3_] in terms of duration and event shape, call for an attempt to classify the observed release events into two different categories. The first category shall contain the short-termed puffs, observable for all concentrations of IP_3_. The other category shall contain the long events showing low channel activity tails and termination by IP_3_ -unbinding, which we identify as waves. A simple classification criterion for the events is given by their potential to trigger another event at a close-by, imaginary cluster, i.e. their potential to support a Ca^2+^ release wave. Therefore, we classify a release event as wave if it induces a local [Ca^2+^] of at least 0.25 µM (corresponding to the dissociation constant of activation, 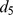, see Table 1) in the entire domain. Otherwise the event is classified as puff. For a detailed discussion of the classification criterion please refer to the supplemental information.

In Fig. 2 G, waves are indicated by a shaded background. Interestingly, the Ca^2+^ signals between 250 s and 275 s in Fig. 2 G clearly differ from the homogeneous signals produced by waves. With the above wave criterion, this burst of channel openings is identified as a sequence of distinct puffs.

**Figure 6:**
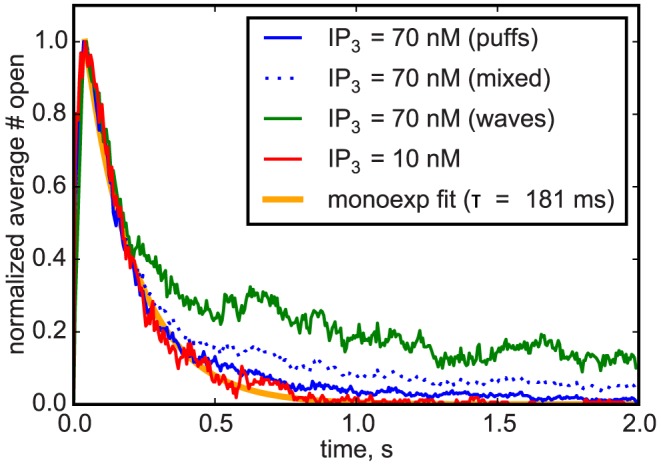
Average event shapes of waves and puffs. To compare behavior at different [IP_3_] concentrations, peaks were normalized to 1. For high [IP_3_], event classification into waves and puffs nicely separates the termination mechanisms into fast inhibition for puffs, and a mix of inhibition and slow IP_3_ inactivation for waves.

To compare the temporal structure of the release events, we averaged the time courses of the number of open channels over all events for each value of [IP_3_]. We then normalized the average time courses for different [IP_3_] to a peak value of 1. Fig. 6 displays the average time courses for 10 nM and 70 nM concentrations. The solid red line shows the resulting average puff for small IP_3_ concentration. The fact, that for [IP_3_] = 10 nM we solely observed puffs, is reflected by a simple exponentially decreasing average number of open channels representing puff termination by Ca^2+^ inhibition on a fast time scale. However, the dotted blue line shows that for large IP_3_ concentration an increased second release phase appears, while for times below 200 ms no deviation from the profile of puffs occurs. Furthermore, Fig. 6 presents averaging of the same events for 70 nM concentration of IP_3_, but now with two groups separating into puffs and waves according to our criterion described above. The solid blue (puffs) and green (waves) curves indicate that our classification between puffs and waves worked out well, as the average event shape for puffs at [IP_3_] = 70 nM shows a decay very similar to that of puffs for [IP_3_] = 10 nM. Analyzing the event shape for waves, we can clearly distinguish the two termination mechanisms: the first 200 ms are dominated by inhibition, while afterwards the slow IP_3_ unbinding takes over.

**Figure 7:**
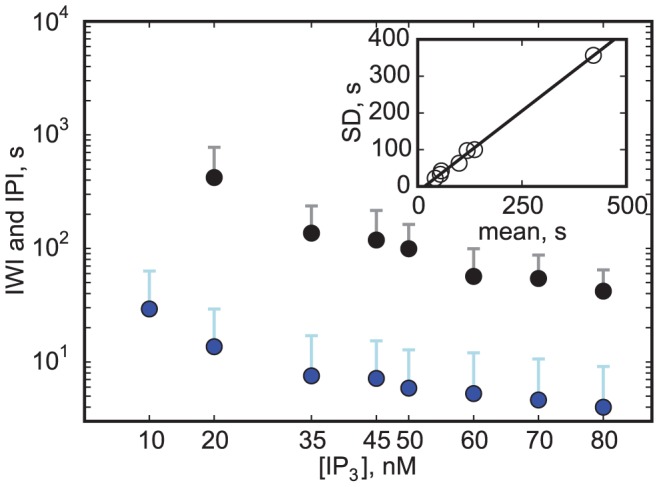
Average inter wave interval (IWI, black) and inter puff interval (IPI, blue) for different [IP_3_]. For [IP_3_] = 10 nM, no waves could be observed. An IPI is defined as time between the end of the preceding and the beginning of the succeeding puff. If two puffs were split by a wave, the IPI was excluded. Error bars denote standard deviations. Inset: Similar to data from [52] there is a clear linear correlation between the average and standard deviation (SD) of the IWI. The resulting coefficient of variation is decreasing from 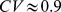 for [IP_3_] = 0.01 µM to 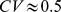 for [IP_3_] = 0.08 µM. The slope of the regression line is 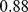, the minimal average IWI (i.e., the intersection with the *x*-axis) is 17 s.

A prominent feature of our model for large [IP_3_] is that here puffs and waves coexist. Occurrences of waves or global oscillation require the interaction of several clusters, and this interaction may affect dynamical aspects of the release, including oscillation period and variability [53]. Nonetheless, consideration of local dynamics generally allows revealing insights into the global aspects and the local dynamics often keep a dominating influence in many oscillating systems. Thus, having distinguished waves from puffs, we now interpret the repeated occurrence of wave-like events as a slow oscillation in the local dynamics and identify the period of global oscillations by the IWI in our simulations. Fig. 7 shows averages for IWI and inter puff interval (IPI) depending on [IP_3_]. Similar as shown by experiments [11], our model predicts decreasing IWI for increasing [IP_3_]. The wave periods are generally in the same range as the periods of global oscillation measured in various cell types [11], [12], [52]. Recent experiments by Thurley et al. [55] showed exponential dependence of average period of global oscillations with stimulation, which is consistent with the scaling of IWI data in Fig. 7. Furthermore, there is a linear relation between the average and standard deviation of the IWI in our simulation results (inset). The slope of the regression line is 0.88 in simulations, which is similar to what was found for several cell types including astrocytes [26]. For HEK cells [26], [55], Hepatocytes [54] and *Xenopus* oocytes [4], [13], smaller variability was measured. This can possibly be understood from more complex effects of coupling, where synchronization can cause higher regularity of periodic dynamics [53].

### Modulated dynamics during IWIs and comparison to experiments

**Figure 8:**
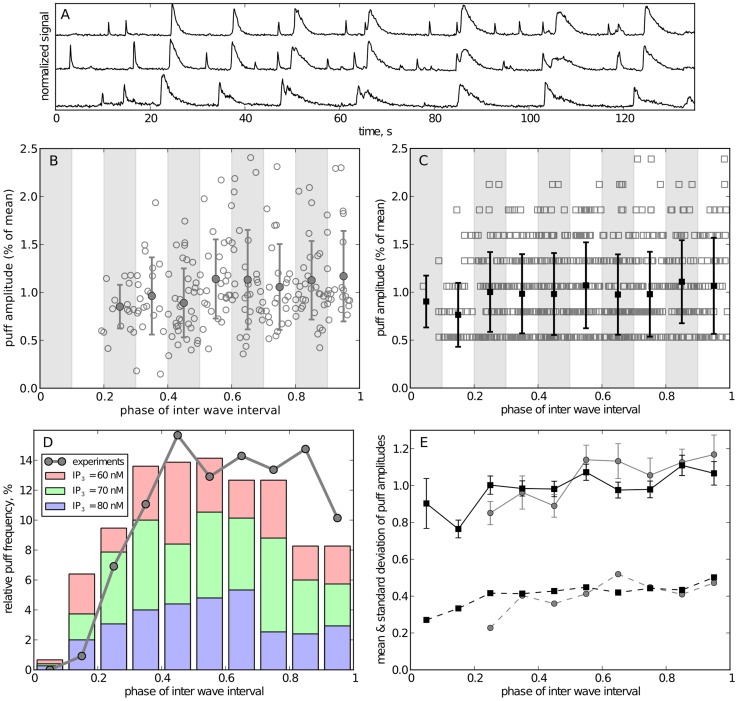
Comparison of inter-wave dynamics for experiments and simulations. (A) shows representative fluorescence traces from 3 puff sites in *Xenopus* oocyte. Waves were identified as large signals that occurred approximately at the same time at all cluster sites. (B, C): scatter plot of puff amplitudes in experimental (B) and simulated traces (C). The amplitude of a puff is defined by the peak fluorescense for experimental data and the peak number of open channels for simulated data. The horizontal axis is the inter-wave phase with 0 corresponding to the time of the preceding wave and 1 corresponding to the time of the next wave. Experimental data was pooled from four experiments, simulation data was pooled from traces for 60, 70, and 80 nM [IP_3_]. The amplitudes of experimental and simulated data were then rescaled to their respective mean amplitude. The data was grouped into 10 intervals (shaded regions); filled markers and error bars denote the mean and standard deviation of each group, respectively. (D) Temporal distribution of puff frequency between two waves after binning of data in 0.1 phases. (E) Mean (solid lines with standard error) and standard deviations (dashed lines) of binned amplitudes for simulations (black) and exeriments (gray).

In [11], [24] it was shown that in *Xenopus* oocytes puffs appear in the phase between succeeding global waves. Exemplary fluorescence traces from experiments with this cell type are shown in Fig. 8A. Here a wave can be identified as a group of large events that appear within temporal proximity at neighboring cluster sites. The remaining events are then identified as puffs and their amplitude has been obtained as peak fluorescence level from traces such as shown in the figure. These amplitudes are plotted in Fig. 8B versus the phase during the IWI, where the phase has been defined as the ratio of time that has passed since the last wave and the total difference between preceding and next wave. Fig. 8C shows for comparison the corresponding puff events from numerical simulations. As seen from Fig. 7, the three data sets for high [IP_3_] are quite similar, suggesting to pool them for better statistics. It is evident from the two plots, that events are absent at the very early phase after waves and that large-amplitude events occur only towards the end of the interval.

To assess quantitatively the data, we have statistically evaluated Fig. 8B, C by pooling puffs from phase intervals of 0.1 and determining the number of events for each bin as well as the mean and standard deviation of their peak amplitudes. The connected dots in Fig. 8D show clearly that the frequency of puffs in experiments is strongly diminished in the early time after each wave. Similarly, we can calculate the temporal distribution of puffs in between waves from simulations. The bars in Fig. 8D are a stacked histogram of those phases for all data sets with high [IP_3_]. From what was observed above, in our model a wave will likely result in a transiently unactivatable cluster with few IP_3_ bound (compare Fig. 2H and 5B). Thus, there is a refractory time where no puffs can arise, which lasts to about 30% of the IWI.

The decrease of puff density at the last two bars at the end of the IWI is likely caused by the fact, that a wave also needs some preceding “silent” phase where the accumulation of activatable channels is not interrupted and partially reset by puffs. A similar decrease of puff frequency just before the wave onset is present in the experimental data. It is more clearly seen for cells with moderate and large IWI, as evident from Fig. 3 in [11].

We finally compare the evolution of puff peak amplitudes for both experimental and simulation data (Fig. 8E). There is very good agreement for behavior of amplitudes (solid lines) and standard deviations (dashed lines). The refractory effect is much smaller for the amplitude of puffs than for their frequency. However, there is a noticeable increase in standard deviation from close to 0.2 to 0.5. More importantly, there is a small number of large amplitude events that are absent in the first half of the IWI but are present in the second half (see B and C). These events represent the large elementary releases that possess the potential to synchronize to a global release wave.

## Discussion

In this paper we have modeled Ca^2+^ release from clusters of intracellular channels where unbinding of IP_3_ occurs during release. The possibility of such unbinding appearing even if surrounding IP_3_ concentrations seem saturating was discovered recently in numerical simulations using a very simple model of spatial coupling of channels [27]. While we have used the mean-field description of Ca^2+^ concentration within the cluster in the prior publication, we here use the FEM with a locally refined spatial grid and adaptive time steps to accurately calculate the complex spatio-temporal Ca^2+^ and buffer distributions. In the present publication we focus on the changes of release dynamics and the role of IP_3_ unbinding for different IP_3_ concentrations.

Our main finding is that there is a complex dynamics in the fraction of subunits bound to IP_3_. The character of this dynamical variation depends on the IP_3_ concentration. For small [IP_3_] there are relatively rare and asynchronous unbindings during the active release phase (i.e., during puffs) so that IP_3_ unbinding contributes little to termination of puffs, puff dynamics and lifetime. Typically, during a puff at most one channel becomes unactivatable because of IP_3_ loss. Nevertheless, the frequent occurrence of puffs leads to accumulated unbinding, so that the number of activatable channels is much smaller than the number of channels in the cluster. Additionally, because of the stochastic nature of unbinding and rebinding, there is a variability of the number of activatable channels at the beginning of each puff. As a result, the dynamics of IP_3_ loss and rebinding modulates the number of channels that open during a puff, which may contribute significantly to the puff amplitude variability observed in recent experiments [31], [33]. Note, however, that in recent studies of puffs cells were loaded with EGTA, which suppresses residual domains and thus affects IP_3_ binding. Therefore, comparison of our current simulations to these findings is not straightforward.

The IP_3_ dynamics at large [IP_3_] is very different from that observed at small [IP_3_]. Most importantly, we find a fraction of release events that last much longer than the typical puff. These events exist because, for higher IP_3_ concentration, a larger number of activatable channels in a cluster is sometimes present and hence larger Ca^2+^ domains and a reduced probability of channel closing follow. We further find that many channels synchronously dissociate IP_3_ during the long-lasting release events. It had been shown in [27] that this behavior can be caused by the extended release during long events or waves, during which most channels repeatedly enter the inhibited state. This increasingly exposes the channels to IP_3_ unbinding, so that most of the channels lose IP_3_. This leads to the paradoxical situation, that for larger IP_3_ concentration more channels dissociate IP_3_ than for small concentration of IP_3_. Eventually, the loss of IP_3_ causes termination of release because of reduction of activatable channel numbers over a timescale of several seconds. Subsequently, a few seconds after termination, IP_3_ rebinds to the receptors and appropriates them for the next opening.

It is interesting to note, that, because of growing dissociation, the increase of [IP_3_] from 10 nM to 80 nM is accompanied by only a slight increase of the typical number of activatable channels from 4 to 5 (Fig. 4, inset). However, it is also pertinent that the variability in activatable numbers at the beginning of an event is increasing with [IP_3_] so that for large [IP_3_] sometimes a number of 8 or more channels are available for opening. Statistically speaking, these are the events of long lifetime and a temporal pattern of striking resemblance with global release in *Xenopus* oocytes [10], [24], [30]. Parker and coworkers have shown that for small IP_3_ concentrations typically only a short release event is observed. For larger IP_3_ concentration, some release events consist of two phases: a short high-amplitude spike and a small-amplitude and slowly decaying second release phase, similar to what is observed in our simulations during long-lasting events. The similarities in the temporal evolution gave us the cue to consider sustained release events in our simulations as global releases or waves.

For further comparison with experiments, we need to classify events based on a quantitative criterion and we here use the potential of an event to trigger release in neighboring clusters as that quantity. This enables us to identify the two types of events as global release or waves and as the small Ca^2+^ puffs that occur in between waves. In our model, the occurrence of puffs between waves is modulated by the slow recovery of IP_3_ binding to the receptors. We compare our results in detail to experimental statistics from waves in *Xenopus* oocytes. Both experiments and simulations show almost no puffs in the early phase after each wave, but a strong increase of puff frequency during the IWI. In contrast, the increase of puff amplitudes during the IWI is modest, while a small number of large amplitude events occurs towards the end of the IWI. To our knowledge, we present the first model that exhibits the increase of puff frequency before waves, which is a hallmark of Ca^2+^ stochasticity. It is questionable whether other possible refractory mechanisms, including ER depletion of Ca^2+^, can generate a similar effect.

We here argue that the frequency of global release events derives from the frequency of large events as defined in our model. The fact that in oocytes not every Ca^2+^ puff causes a wave can be immediately understood, since the stochastic variability of Ca^2+^ release events produces only a subset of events that can lead to global release. The resulting period of waves is indeed in the range of experimental observations in many cell types. We have here assumed that the period of waves is set by a single cluster site in the cell that emanates the waves. This assumption may be justified for some cell types, where waves appear repeatedly at focal sites [11]. For other cell types it may be more realistic to assume that every cluster site can initiate a wave and the period of waves is then set by the first long release event in any of the clusters and the period of waves or global oscillation becomes shorter than the IWI of the individual cluster.

At the center of the effects described in this paper is the repeated burst-like opening of channels because of residual Ca^2+^ and the subsequent IP_3_ unbinding during release. How realistic is this scenario? IP_3_ unbinding depends first on the respective dissociation constants for IP_3_ binding, specifically the constants 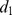 and 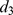. The values of 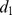 and 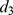 in the DYK model, as obtained from fitting to patch clamp experiments, differ by orders of magnitude, which reflects the fact that the open probability peak of the IP_3_R channel moves to larger Ca^2+^ concentrations with increasing [IP_3_] (see [27] for a detailed discussion). This contrast in dissociation constant was incorporated in many models of Ca^2+^ release. However, since the time a channel spends in the inhibited state is relatively short, it is also necessary for an effect that the unbinding rate 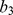 be sufficiently large. Note that this rate constant is not directly observable from experiments and was in the present study chosen to the order of 1 s^−1^ to allow unbinding during long release events (duration 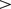
1 s). It should also be noted that IP_3_ unbinding during occupancy of the inhibited state is not a unique property of the DYK scheme. Other models of IP_3_ gating also allow dissociation of IP_3_ from the inhibited configuration. This process presumably reflects the shift of the open probability curve with growing [IP_3_] and should therefore be universal for IP_3_ gating models. This particularly holds for sequential binding models [56] and newer models that are based on channel states and not subunit states [29].

Finally, we would like to suggest further experimental studies that could help to validate our approach. One experimental verification could result from the model's prediction of long duration events even in absence of coupling to other clusters. These long puffs do so far not appear in experiments since addition of exogenous buffer EGTA is used to prevent waves at large IP_3_ concentration. In contrast, our finding could be tested from experiments on genetically engineered cells that only possess one cluster. Further indirect evidence may be obtained from comparison to experiment with repeated stimulations with IP_3_ and Ca^2+^ which were described for oocytes [10] and, more recently, for Purkinje cells [57]. Such experimental protocols require simulations with fixed initial conditions for comparison, which is different from our current focus on long-time simulations.

Another debated issue of calcium oscillations regards the occurrence of concomitant oscillations of free IP_3_ concentrations. It is puzzling that in some cells [IP_3_] oscillates together with [Ca^2+^] while in other cells this is not the case [58]. This [IP_3_] oscillation has been attributed to further metabolic processes, where changes in Ca^2+^ concentration affect the synthesis and degradation of [IP_3_] [59]. However, our model provides an alternative explanation in that for certain situations the unbinding of IP_3_ from the receptors during a wave could lead to larger free IP_3_ concentrations and rebinding to lower concentrations. Quantitative comparison of our predictions of this oscillation mechanism may be possible with a more detailed knowledge of concentrations of IP_3_ receptors in different cell types and provide an explanation for the presence or absence of concomitant [IP_3_] oscillations independent of possible metabolic IP_3_ processes.
